# Colouring of Pacific barkcloths: identification of the brown, red and yellow colourants used in the decoration of historic Pacific barkcloths

**DOI:** 10.1186/s40494-018-0243-9

**Published:** 2019-01-09

**Authors:** T. H. Flowers, M. J. Smith, J. Brunton

**Affiliations:** Centre for Textile Conservation and Technical Art History School of Culture and Creative Arts University of Glasgow, 8 University Gardens, Glasgow, G12 8QH UK

**Keywords:** Pacific barkcloth, Pigments, Dyes, HPLC, XRF, Colorants, Identification

## Abstract

Barkcloth textiles made in the Pacific islands and collected by western explorers in the eighteenth and nineteenth centuries form part of many museum collections worldwide. Here high-performance liquid chromatography (HPLC) and X-ray fluorescence (XRF) were used on cloths that were highly coloured or pigmented specifically focussing on identifying the red, yellow and brown colorants. The cloths studied came from collections held at the Hunterian, University of Glasgow, the Economic Botany Collection, Royal Botanic Gardens Kew and the Centre for Textile Conservation and Technical Art History, University of Glasgow. HPLC analysis was carried out following a sequential extraction procedure to minimise changes to the colorants during extraction. A portable XRF was used so no invasive sampling was required. A small number of plant derived colorants were found, *Morinda citrifolia*, noni (morindin or morindone), *Rubia tinctorum* (madder), tree tannins and *Curcuma longa* (turmeric) plus an inorganic colorant, iron oxide. For 40 samples a single colorant was found while in the remaining 12 samples combinations of up to three colorants were found. Madder was found in only 2 samples on the same cloth. The morindone coloured samples were all red whereas morindin samples were both red and yellow. Morindin was used predominantly in combination with other colouring agents. A combination of iron ochre and organic colorant was found in 4 samples. These findings show that despite the numerous potential colorant sources for red, brown and yellow shades listed in the many accounts of historic barkcloth making, only five types of plant colourant and one inorganic pigment were found. There are a number of potential reasons for these findings. Some colours may have faded and so no longer appear coloured. It is also possible that, as some of these cloths were prepared specifically as gifts for visitors or for ceremonial uses, the makers used materials that they knew would retain their integrity over time. Perhaps, like artisans worldwide, experience had taught them that some colorants, although initially bright and vivid, faded over time.

## Introduction

Barkcloth (tapa) is one of the most distinctive products of cultures originating from the Pacific islands. Barkcloth is a non-woven material made from beaten inner bark and often referred to as a bast fibre. Tapa production was central to providing clothing and bedding, as well as for decoration and ceremonial purposes [1]. The name *tapa,* now a universal term for Pacific barkcloth, derives from the Samoan term *tapa* for the uncoloured border of a piece of barkcloth. In Hawaii *kappa* describes a range of different types of barkcloth. Tapa is constructed from different types of plant, most commonly *Broussonetia papyrifera* (paper mulberry), *Artocarpus altilis* (breadfruit), *Ficus prolia* (banyan) or *Pipturis albidus* (mamaki), producing a matted, strong and fibrous structure, beaten to resemble a paper-like material or textile. Varied approaches to making tapa are noted within different Pacific cultures, but these are variations based on a central methodology [2].

Traditionally, the colourants used to dye plain barkcloth were obtained from plants, trees and earth pigments. When different cultures met through trading, especially in the Tonga–Fiji–Samoa region, different patterns on barkcloth became identifiers of where they originated. The arrival of missionaries and other Westerners during the nineteenth century saw a change in the materials used to produce barkcloth, with imported materials sometimes used.

Whilst the number of different types of plants recorded as being used to provide differing colours is large and varied [3] the literature on the analysis of the various types of dyes found to be present in backcloths remains narrow [4, 5]. A comprehensive review on the materials used in barkcloth production by Larson [6] listed only a few sources of plant colorants and inorganic pigments as commonly used. There has been very little scientific analysis carried out on the colourants on these cloths excepting work by Bisulca et al. [4] and the published literature on the materials used and their manufacture for the most part comes from historic accounts of early missionaries or explorers. Research carried out to determine the colorants used on Hawaiian barkcloth noted that there are over 10 plant species documented as historic red dyestuffs, however from the barkcloth pieces analysed for red colouring all came from noni (*Morinda citrifolia*). In cases where the red design was not organic, the red colourant was found to be an iron oxide pigment [4].

The findings presented here focus on the analysis of organic and inorganic colourants used on cloths and detail extraction protocols which minimise damage to colorants under study and also reduce the number of steps used in the process. In this study cloths from the eighteenth and nineteenth centuries from 3 collections are used to determine the variation and/or similarities of these colourants by carrying out analysis using high-performance liquid chromatography (HPLC) and X-ray fluorescence (XRF) on cloths that were highly coloured or pigmented specifically focussing on identifying the red, yellow and brown colorants.

## Materials and methods

### Barkcloths

The 54 historic cloths analysed came from three collections, The Hunterian, University of Glasgow, Glasgow (33), the Economic Botany Collection (EBC), Royal Botanic Gardens, Kew, London, (16) and a sample book at the Centre for Textile Conservation and Technical Art History, University of Glasgow (5). These are detailed in Table 1. The Hunterian acquired its collection from several collectors including William Hunter who acquired barkcloths collected during Captain Cook’s voyages during the 1760 s and 1770 s. Alexander Angus donated a collection of Hawaiian and Tahitian cloths in 1810 and George Turner lived in Samoa for many years from 1841 and donated his collection. The EBC barkcloths came from a number of donors mainly in the second half of the nineteenth century. A sample book at the CTCTAH states on the first page that the samples within it were ‘collected during the three voyages of Captain Cook to the Southern Hemisphere’ which took place between 1768 and 1780. It consists of 30 samples of various types of cloths. The origins of the cloths given in Table 1 are based on current curatorial records of each collection.

**Table 1:** Details of the size, descriptions and areas of backcloths sampled

Accession number	Origin (tentative)	Length (mm)	Width (mm)	Description (mm)	Colours sampled HPLC	Colours sampled XRF
*CTCTAH sample book*						
CTCSB2016 No.10	Tahiti	209	131	Plain yellow	Yellow	
CTCSB2016 No.18	Tahiti	89	52	Red/yellow/black pattern	Yellow	
CTCSB2016 No.20	Hawaii	71	54	Plain red	Red	
CTCSB2016 No.23	Tahiti	200	128	Plain red	Red	
CTCSB2016 No. 29	Tonga	67	49	Red/yellow/black pattern	Yellow	
*Hunterian museum*						
E417/2	Samoa	2150	1865	Black/yellow pattern on undyed background	Yellow	
E417/5	Fiji	2200	590	Black with red border	Red	
E417/11	Tonga	1570	710	Red zigzags on black background	Red	Red
E457/3	Hawaii	1650	1350	Red and yellow stripes on undyed background	Yellow	
E458/1	Samoa or Cook Islands	1840	900	Yellow/black pattern on undyed background	Yellow	
E458/2	Hawaii	950	750	Thin red stripes on yellow background	Yellow	
E458/3	Fiji	2120	1800	Brown line pattern on undyed background red/black border	Brown, red	
E458/4	Tahiti or Marquesas Islands	3930	1480	Plain red-brown	Red-brown	
E458/6	Tonga	3530	1580	Plain brown and brown/yellow pattern	Brown	
E591/4	Polynesia	1220	950	Undyed cloth	Undyed	
E594/2	Tahiti	1310	430	Undyed cloth	Undyed	
E594/8	Tahiti	810	360	Undyed cloth	Undyed	
E595	Tonga	2930	1590	Red with undyed spots	Red	Red
E595/1	Tahiti	1960	1090	Red pattern on yellow background	Red, yellow	
E596/1	Tahiti or Hawaii	440	250	Undyed cloth		Undyed
E596/5	Tahiti	415	290	Undyed cloth		Undyed
E596/6	Tahiti	300	140	Undyed cloth	Undyed	
E596/7	Fiji	1550	954	Undyed cloth		Undyed
E596/8	Tahiti	173	153	Undyed cloth		Undyed
E598/1	Hawaii	233	172	Red/yellow/black/undyed stripe pattern	Red, yellow	Red, yellow, undyed
E598/2	Hawaii	198	160	Red/black pattern	Red	Red
E598/3	Hawaii	443	113	Red/yellow/black pattern	Red	Red
E598/4	Hawaii	350	247	Red/yellow/black pattern	Red	Red
*Hunterian museum*						
E599	Tahiti or Hawaii	1300	1050	Dark brown flaking coating	Dark brown	Dark brown, bare
E600	Tahiti or Hawaii	945	440	Red border stripe on undyed cloth	Red	Red, undyed
E601	Hawaii	910	755	Red, grey and undyed stripes	Red, undyed	
E602	Hawaii	780	500	Red/yellow/black pattern	Red, yellow	Red, yellow
E603	Tahiti	1100	670	Plain red-brown cloth	Red-brown	
E606	Cook Islands	522	175	Plain red and undyed blocks	Red, undyed	Red, undyed
E608	Tahiti	530	510	Plain yellow	Yellow	
E610	Fiji	1545	890	Undyed cloth		Undyed
E611/3	Hawaii	2100	1200	Mottled grey		Grey
E667	Hawaii	990	860	Red/yellow/black pattern	Red	
*Kew EBC*						
42853A	Hawaii	110	85	Plain red cloth	Red	Red
42861	Samoa	2340	950	Poncho red/black/undyed pattern		Red, undyed
42863	Samoa	2045	1950	Stripe and leaf pattern on undyed background	Red-brown, Yellow	
42885H	Hawaii	560	230	Plain dark brown cloth	Dark brown	
42947A	Hawaii	2190	955	Red handprints on yellow background	Red, yellow	
42947B	Hawaii	1550	1340	Red stripes on yellow background	Red, yellow	Red, yellow
42958A	Hawaii	1760	1713	Red pattern on yellow background	Yellow	
42958B	Hawaii	1350	1180	Brown and fine red pattern on undyed background	Brown, light brown	
42958C	Hawaii	1374	938	Red leaf pattern on yellow background	Red, yellow	
42965	Hawaii	2732	2117	Undyed cloth	Undyed	
42966	Hawaii	3455	2340	Mottled red on undyded background	Red	
42967	Hawaii	3308	2576	Red/purple mottles in triangular pattern	Red, purple	
42979	South Sea Islands	1983	1905	Plain glossy red coating	Red	
67802A	Unknown	159	133	Red/yellow stripe pattern	Red	Red
73329	Tahiti	2340	1570	Brown pattern/tassles on undyed background	Brown	
98041	Unknown	2915	945	Plain red-brown	Red-brown	Red-brown

### Extraction and HPLC analysis

Samples were taken from the edges of cloths or from damaged areas, from regions of uniform colour. Not all colours could be sampled if they did not appear at edges or the areas were too small or the cloths themselves were small. Depending on the availability of material 1–5 mg samples were used for HPLC analysis. Because the colorants frequently only lightly coat the surface of the barkcloth fibres the weight of cloth sampled is not a good indicator of the weight of dye present.

The extraction method was based on the findings of Wouters et al. [7] who investigated the use of mild extractants for textiles and paints to minimise changes to the dyes during extraction. Acetone was omitted from their mildest extractant (oxalic acid/methanol) to eliminate the need to evaporate the extract to dryness and redissolve to prevent interference in the UV detection by acetone. For more acidic extractions we substituted hydrofluoric acid–methanol with hydrochloric acid–methanol on safety grounds. A sequential extraction procedure was used to enable extractants of increasing acidity to be used with a single barkcloth sample.

The tapa sample (1–5 mg) was placed in a 500 µL polypropylene microcentrifuge tube and 250 µL of the initial extracting solution added. A small hole was made in the tube lid to prevent pressure build up and the tube placed in a water bath as follows. Extraction 1: 60% methanol 0.002 M oxalic acid 80 °C 20 min; Extraction 2: 50% methanol 0.1 M HCl 80 °C 20 min; Extraction 3: 50% methanol 1 M HCl 90 °C 30 min. After each extraction the extract was made back up to the nominal volume with the appropriate extracting solution and filtered through a 4 mm 0.2 µm Teflon syringe filter into a 300 µL fixed insert autosampler vial. A further 50 µL of extracting solution was used to rinse the tube and filter into the autosampler vial. The sample was then extracted with the next extracting solution.

Analysis was carried out using a gradient high pressure chromatography (HPLC) system with a UV diode array detector. The Merck-Hitachi HPLC system comprised: L-7200 autosampler, L-7100 gradient pump, Jones Chromatography Genesis C18 4 µm 250 by 4.6 mm column, L-7350 column oven, L-4500 diode array detector and L-7000 HPLC system manager software. The extracts were analysed using acetonitrile:water gradient elution (30% to 98% acetonitrile over 30 min) with 0.1% phosphoric acid (Table 2). Spectra were collected from 200 to 600 nm and a chromatogram extracted at 425 nm. Where tannins were present a second HPLC gradient was used (10% to 95% acetonitrile over 25 min) with 0.25% phosphoric (Table 2). Spectra were collected from 200 to 600 nm and a chromatogram extracted at 275 nm. 59 samples were tested from 47 cloths (Table 1).

**Table 2:** HPLC gradients

Time (min)	Water (%)	Acetonitrile (%)	5% H_3_PO_4_ (%)
*HPLC gradient 1*			
0	68	30	2
25	0	98	2
30	0	98	2
30.1	68	98	2
35	68	98	2
*HPLC gradient 2*			
0	85	10	5
25	0	95	5
25.1	85	10	5
30	85	10	5

Components were identified primarily on the basis of their UV–visible spectra and retention time. A subset of barkcloth sample extracts were analysed using a Shimadzu LC2010A HT LC system and LCMS2010EV Mass spectrometer HPLC–MS system using the same Genesis column and water:acetonitrile gradient 1 with 0.1% formic acid. UV detection was at 370 nm and MS detection was in negative ion mode to confirm the UV based component identity by the mass of the molecular ion. A soft ionisation was used to preserve the molecular ion.

Because of the scarcity of available standards to generate reference UV–visible spectra and retention times, an in-house spectrum library was generated using methanol extracts of plant materials. Peak identification was based on literature references of the major components present, their relative retention characteristics and their UV–visible data [5]. Confirmation of peak identity was obtained from the mass of the molecular ion using LC–MS.

### XRF analysis

XRF analysis was carried out using a Niton XL3t GOLDD + handheld XRF in mining mode (Main range 15 s, Low range 15 s, High range 10 s, Light range 20 s). XRF analysis could be carried out anywhere on a cloth but required a uniform area with a minimum diameter of 4 mm. 28 samples (9 undyed controls, 19 dyed samples) were tested from 21 cloths (Table 1).

## Results and discussion

### HPLC

**Fig. 1:**
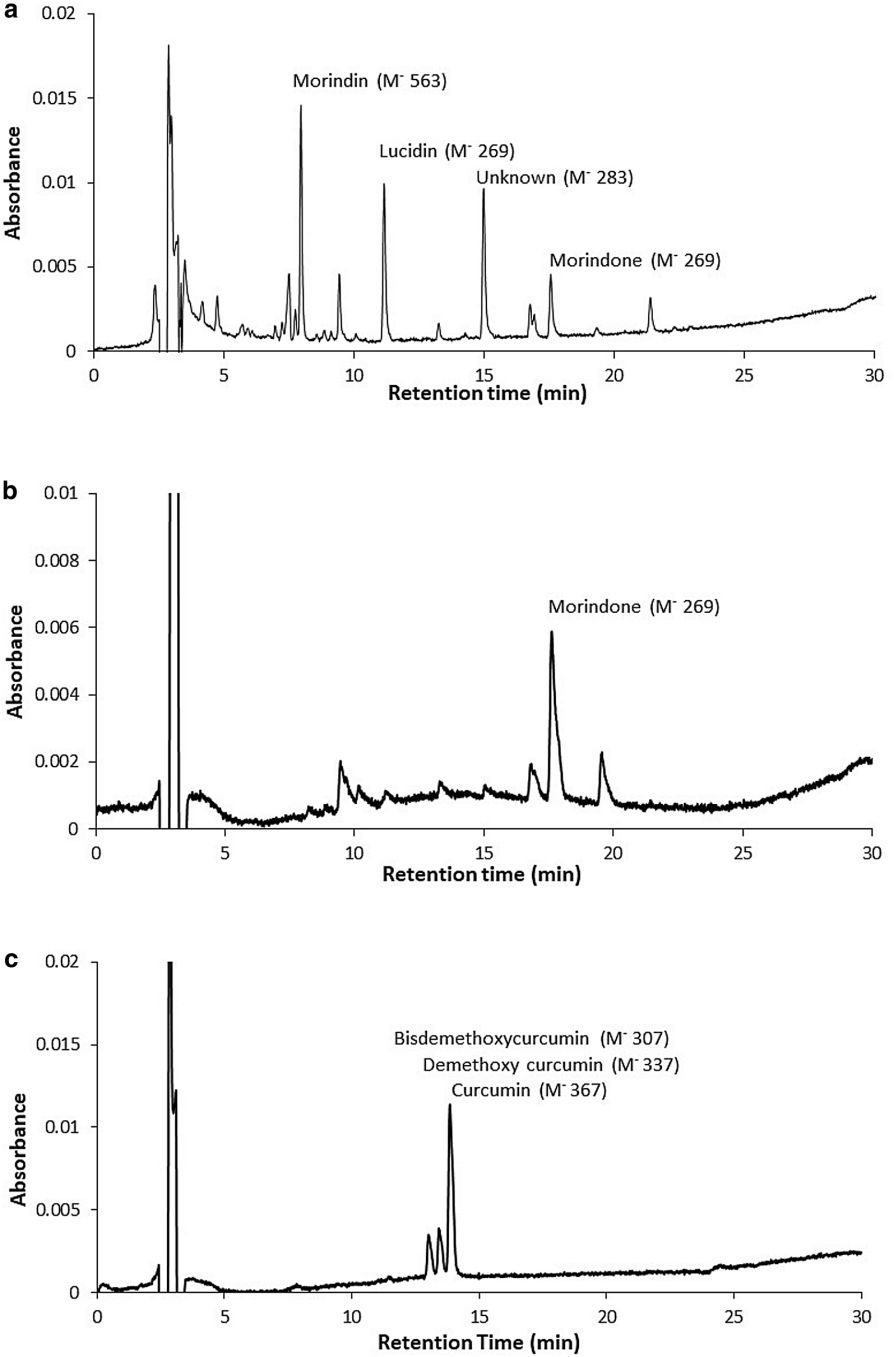
Typical chromatograms of barkcloth sample extracts. **a** Untreated noni; **b** treated noni; **c** turmeric

Seven very light yellow/brown barkcloth samples all yielded chromatograms with no peaks suggesting that this represents the varying colours of the undyed barkcloth. A further two similarly coloured samples showed the presence of turmeric. The 49 coloured samples where chromatographic peaks were obtained produced organic dyes from 4 plant sources: noni (*Morinda citrifolia*), turmeric (*Curcuma longa*), madder (*Rubia tinctorum*) and non-specific tannins. Identification of the plant sources was based on the presence of their major characteristic peaks. In the case of noni it is generally accepted that the presence of morindin and/or morindone is sufficient [5] even though the mixture of anthraquinones present as minor constituents may be more complex. Noni was found in two forms, dominated by either the glycoside morindin (Fig. 1a shows a typical chromatogram) or the aglycone morindone (Fig. 1b). Many of the anthraquinone plant dyes are present in the plant as glycosides where the coloured anthraquinone molecule is bound to one or more sugar molecules. During dye manufacture the glycosides can be hydrolysed by heat or acid or by the enzymes present in the plant extract. The noni based dyes (morindin and morindone) are naturally yellow but the colour is pH dependent, red above pH 10 [8], and burnt lime can be used to create a red dye [6]. Strongly acid extracting solutions can hydrolyse the glycosides releasing the aglycone anthraquinone and further degrade the molecule thus losing information on the exact nature of the dye molecule and the dye manufacturing process [9]. A review paper by Degano et al. [10] discusses the effects of acidic methanol used for the extraction of dyes in historic paint and textiles samples. Weaker acid extractions using oxalic acid or hydrofluoric acid have been recommended for paint samples to avoid hydrolysis of the dye molecules [7, 11]. The dyes used on barkcloths are frequently present as a surface coating on the barkcloth fibre without use of a mordant and are not strongly bound to the fibres. A weakly acid extracting solution can successfully extract many of the dyes while preserving the glycosides and the information they provide. A sequential extraction procedure with increasing acidity allow extraction of labile weakly bound dyes as well as the more strongly bound dyes.

**Fig. 2:**
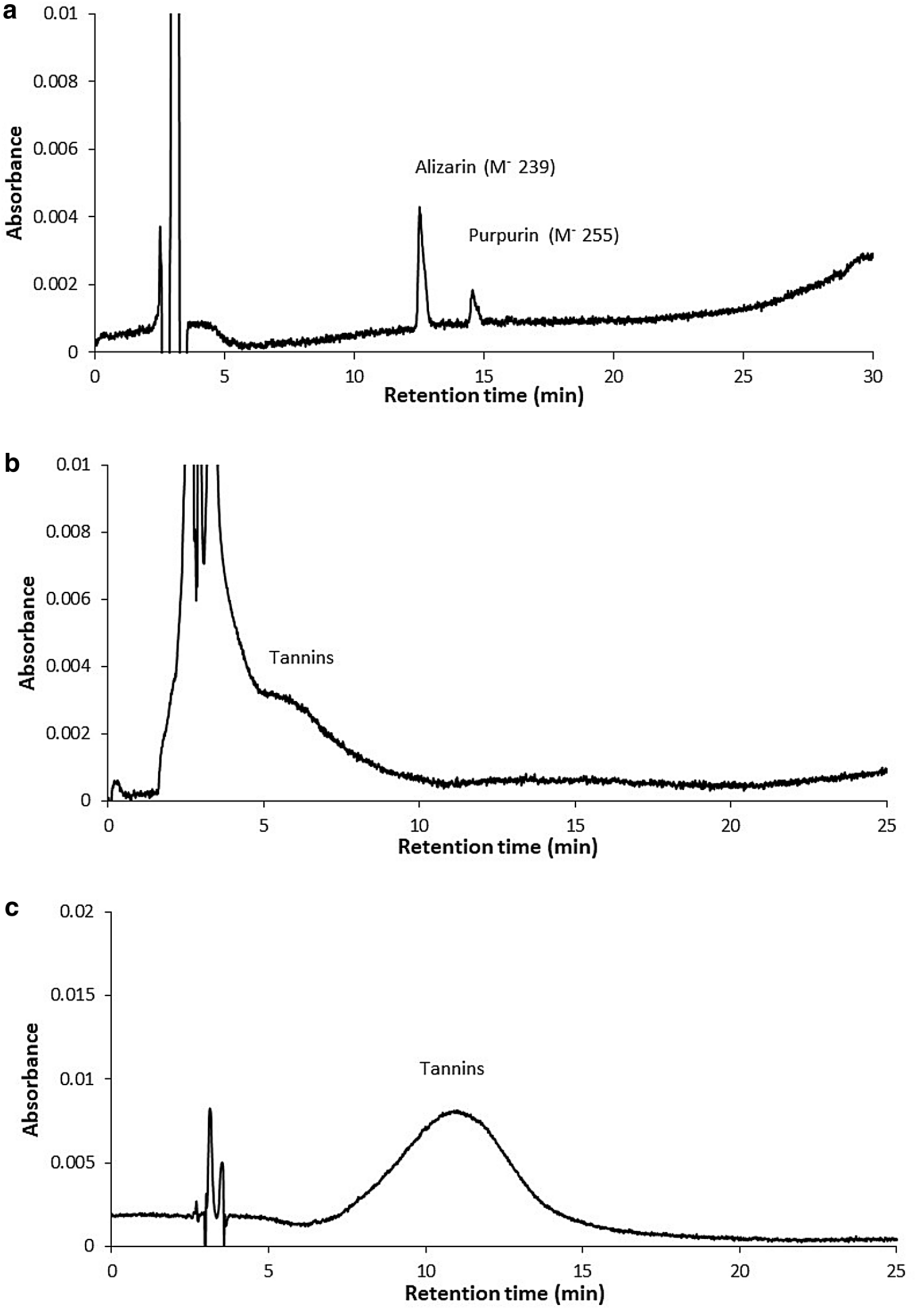
Typical chromatograms of barkcloth sample extracts. **a** madder; **b** tannins using HPLC gradient 1; **c** tannins using HPLC gradient 2

The chromatogram of turmeric showed 3 characteristic peaks: bisdemethoxycurcumin, demethoxycurcumin and curcumin (Fig. 1c). Some cloths coloured with turmeric which had faded with age often appeared similar in colour to the undyed cloths but these 3 compounds could be detected. The madder chromatogram showed peaks for alizarin and purpurin (Fig. 2a). The tannins produced a broad shoulder on the injection noise at about 4 min (Fig. 2b) which was resolved into a broad peak at about 10 min using HPLC gradient 2 (Fig. 2c). In the 1 M HCl extracts there was some hydrolysis to produce anthocyanidins but it was not possible to fingerprint the plant source. A more detail examination of the tannins could perhaps be achieved by digesting the extracts to hydrolyse the tannins or by use of a different chromatographic column. Tamburini et al. [5] also used a C18 column but it has been reported that an amide column [12] might be more effective. This was, however, beyond the scope of this work.

**Fig. 3:**
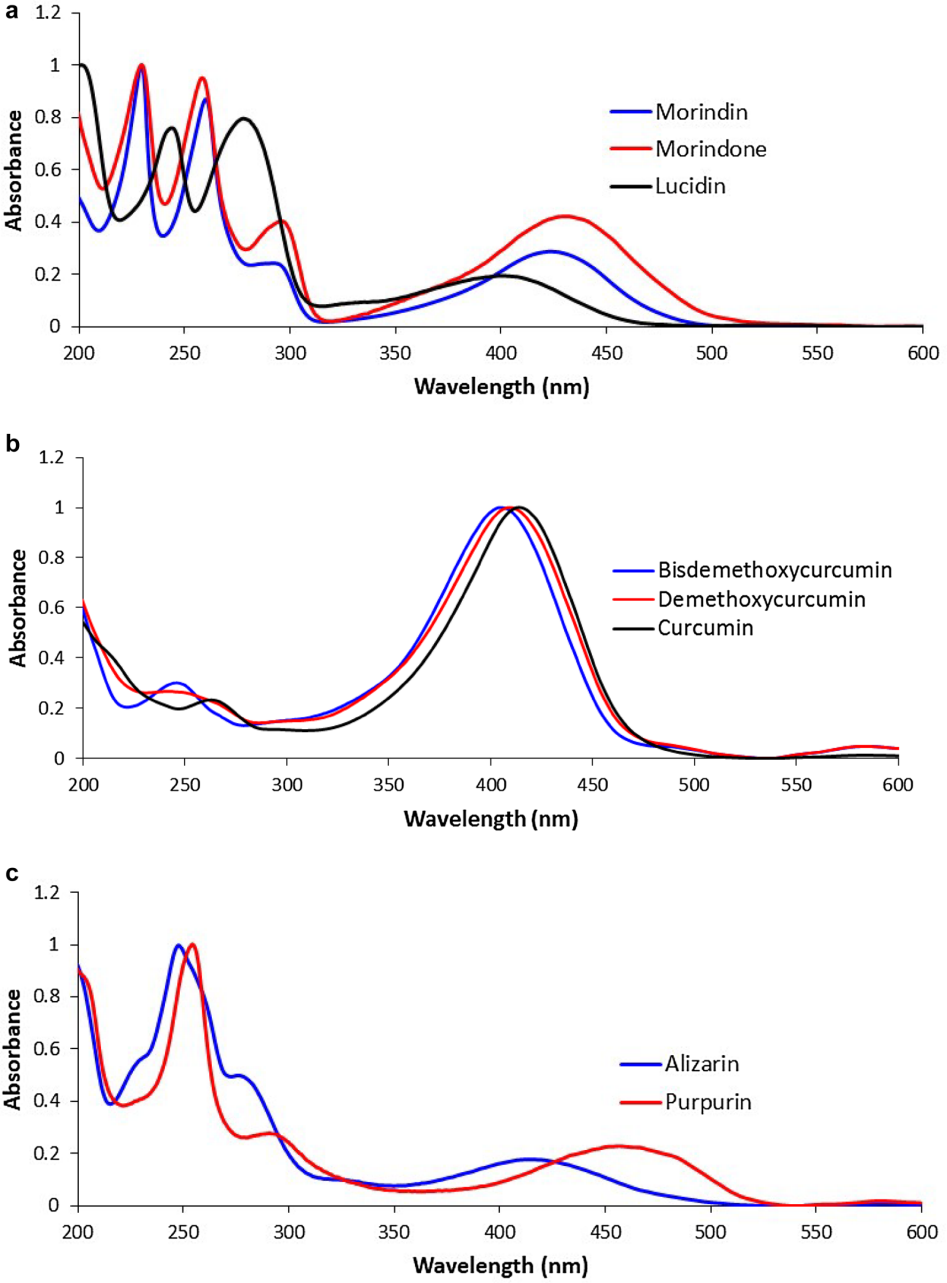
UV–Vis spectra of **a** major noni components; **b** turmeric components; **c** madder components

Morindin, morindone, curcumin, demethoxycurcumin, bis demethoxycurcumin and tannins were readily extracted in the first oxalic acid:methanol extraction. Alizarin and purpurin showed most clearly in the 0.1 M HCl:methanol and and M HCL:methanol extractions. UV–visible spectra of these components are shown in Fig. 3. The identity of these components was confirmed by HPLC–MS using the mass of the negatively charged molecular ion.

The use of the milder method extraction 1 prevented the decomposition of glycosides allowing for a better understanding of the how these colourants were prepared for use. This suggests that the noni root was prepared in different ways for barkcloth decoration although no correlation to the island of origin was observable in this study. The ease of extraction of most of the colorants points to the lack of traditional metallic mordenting.

### XRF

The results are shown in Table 3. Iron oxide was the only inorganic pigment detected and elevated titanium was associated with the high iron content. The 9 undyed control samples all showed low undetectable background Fe levels < 0.002%. Of the 19 coloured samples tested 13 were negative and 6 positive for iron. Of these 6 samples 4 also had an organic pigment present (2 tannin and 2 traces of turmeric) and only 1 contained iron alone. The poncho Kew 42861 (Fig. 5c) could not be sampled for HPLC extraction. Calcium was present in both coloured and uncoloured cloths up to 1.7%. These levels could be due to the manufacturing process where some cloths would have been polished using shells (calcium carbonate) or inherent in the bark fibres. Burnt coral (calcium hydroxide) can be used to produce the red dyes from noni but XRF would not distinguish the form of calcium.

**Table 3:** Results of XRF analysis

ID No		Al (%)	Si (%)	P (%)	S (%)	Cl (%)	K (%)	Ca (%)	Ti (%)	Fe (%)
*Coloured*										
E417/11	Red	0.63	2.29	0.21	0.15	0.34	0.69	0.57	0.939	1.773
E595	Red	5.28	6.64	0.08	0.06	0.32	0.47	0.46	0.272	4.172
E598/1	Red	0.19	0.76	0.27	1.30	1.13	0.41	0.97	0.017	0.053
E598/1	Yellow	0.21	0.70	0.18	1.22	1.02	0.47	0.94	0.018	0.049
E598/2	Red	0.26	1.14	0.38	1.28	0.75	0.42	0.92	0.016	0.057
E598/3	Red	0.17	0.62	0.24	1.14	1.17	0.21	1.03	0.013	0.033
E598/4	Red	0.19	0.77	0.31	1.07	0.84	0.34	1.23	0.010	0.017
E599	Dark brown	0.12	0.41	0.11	0.23	0.06	0.04	1.62	0.046	0.333
E600	Red	0.18	0.60	0.30	0.81	0.30	0.04	1.10	0.021	0.046
E602	Red	1.08	3.04	0.50	0.52	0.39	0.45	0.44	0.330	1.031
E602	Yellow	0.57	1.71	0.38	0.49	0.69	0.38	0.46	0.193	0.602
E606	Red	< LOD	0.29	0.08	0.50	0.06	0.11	1.15	< LOD	< LOD
E611/3	Grey	< LOD	0.07	0.03	0.33	0.97	0.05	0.46	< LOD	< LOD
42853A	Red	0.11	0.22	0.17	0.95	1.37	0.21	0.69	< LOD	< LOD
42861	Red	0.42	1.37	0.24	0.23	0.18	0.58	0.39	0.459	1.149
42947B	Red	< LOD	0.06	0.15	0.29	0.44	1.00	1.69	< LOD	< LOD
42947B	Yellow	< LOD	< LOD	0.16	0.16	0.37	1.08	0.99	0.005	< LOD
67802A	Red	< LOD	0.31	0.19	0.42	1.13	0.12	1.74	< LOD	< LOD
98041	Red-brown	< LOD	0.07	0.07	0.24	0.03	1.01	1.32	< LOD	< LOD
*Uncoloured*										
Average (n = 9)		0.02	0.26	0.04	0.53	0.29	0.10	0.55	0.003	0.006
Max		0.190	0.858	0.289	1.499	1.185	0.463	1.042	0.022	0.048
Min		< LOD	< LOD	< LOD	0.026	0.029	< LOD	0.102	< LOD	< LOD
*LOD*		0.057	0.034	0.013	0.020	0.008	0.021	0.051	0.005	0.010

The degree of weathering of the ochres indicated by the ratios of immobile elements (Al and Ti) to more mobile elements (Si and Fe) might be useful in identifying the sources of the iron ochre [13] but would require a much larger dataset than here.

### Analysis summary

A table of all the cloths and the colourants found on them is shown in Table 4 and a summary of all the coloured cloths is shown in Table 5. From all the analysis carried out on the coloured cloths listed in Table 4 a small group of 5 plant derived colourants and 1 inorganic pigment were all that were found. For 40 samples a single colourant was found while in the remaining 12 samples a combination of up to three colourants were detected. Madder was found in only two samples on the same cloth. The noni (morindone) samples were all red whereas the noni (morindin) were both red and yellow. Noni (morindin) was used predominantly in combination with other colouring agents. A combination of iron ochre and organic dyes was found in 4 samples. The ease of extraction of most of the colorants points to the lack of traditional metallic mordenting. However, historically tree tannins were used as mordents [14]. Our findings show (Table 5) that although tannins were found as a single colorant in 11 cloths they were also found in combination with other colorants in 9 cloths where they may have been acting as a mordent.

**Table 4:** Colorants found by HPLC and XRF

Accession number	Description	Colours sampled	Colourant found
*CTCTAH sample book*			
CTCSB2016 No. 10	Plain yellow	Yellow	Turmeric
CTCSB2016 No. 18	Red/yellow/black pattern	Yellow	Turmeric
CTCSB2016 No. 20	Plain red	Red	Noni(T)
CTCSB2016 No. 23	Plain red	Red	Noni(T), tannin
CTCSB2016 No. 29	Red/yellow/black pattern	Yellow	Turmeric
*Hunterian museum*			
E417/2	Black/yellow pattern on undyed background	Yellow	Turmeric
E417/5	Black with red border	Red	Tannin
E417/11	Red zigzags on black background	Red	Tannin, iron oxide
E457/3	Red and yellow stripes on undyed background	Yellow	Turmeric
E458/1	Yellow/black pattern on undyed background	Yellow	Noni(U)
E458/3	Red/brown/black pattern	Light brown	Tannin
		Red	Tannin
E458/2	Thin red stripes on yellow background	Yellow	Tannin
E458/4	Plain red-brown r	Red-brown	Tannin
E458/6	Plain brown and brown/yellow pattern	brown	Tannin
E595	Red with undyed spots	Red	Iron oxide
E595/1	Red pattern on yellow background	Red	Noni(U), tannin
		Yellow	Noni(U)
E598/1	Red/yellow/black/undyed stripe pattern	Red	Noni(T)
E598/2	Red/black pattern	Red	Noni(T)
E598/3	Red/yellow/black pattern	Red	Noni(T)
E598/4	Red/yellow/black pattern	Red	Noni(T)
E599	Dark brown flaking coating	Dark brown	Tannin, iron oxide
E600	Red border stripe on undyed cloth	Red	Noni(T)
E601	Red, grey and undyed stripes	Red	Noni(T)
E602	Red/yellow/black pattern	Red	Iron oxide, trace turmeric
		Yellow	Iron oxide, trace turmeric
E603	Plain red-brown cloth	Red-brown	Tannin
E606	Plain red and undyed blocks	Red	Tannin
E608	Plain yellow	Yellow	Turmeric
E667	Red/yellow/black pattern	Red	Noni(T)
*Kew EBC*			
Kew 42853/2	Plain red cloth	Red	Noni(T)
Kew 42861	Poncho red/black/undyed pattern	Red	Iron oxide
Kew 42863	Multi-coloured stripe and leaf pattern on undyed background	Red-brown	Turmeric, tannin
		Yellow	Turmeric
Kew 42885	Plain dark brown cloth	Dark brown	Tannin
Kew 42947/1	Red handprints on yellow background	Red	Noni(U), tannin
		Yellow	Turmeric
Kew 42947/2	Red stripes on yellow background	Red	Noni(U)
		Yellow	Turmeric
Kew 42958(1)	Red pattern on yellow background	Yellow	Turmeric
Kew 42958(2)	Red/brown pattern on undyed background	Brown	Noni(U), tannin
		Light brown	Noni(U)
Kew 42958(3)	Red leaf pattern on yellow background	Red	Noni(U), tannin, turmeric
		Yellow	Noni(U), turmeric
Kew 42966	Mottled red on undyed background	Red	Noni(T)
Kew 42967	Red/purple mottles in triangular pattern	Red	Madder
		Purple	Madder
Kew 42979	Plain glossy red coating	Red	Tannin
Kew 67802	Red/yellow stripe pattern	Red	Noni(T)
Kew 73329	Brown pattern on undyed background, brown tassles	Brown	Noni(U), tannin
Kew 98041	Plain red-brown	Red-brown	Tannin

**Table 5:** Summary of colorants found in all barkcloth samples

Colorant	Number of barkcloth samples
*Single*	
Madder	2
Noni(morindin)	4
Noni(morindone)	11
Tannin	11
Turmeric	10
Iron oxide	2
*Combination*	
Noni(morindin) tannin	4
Noni(morindone) tannin	1
Noni(morinidin)turmeric	1
Noni(morindin) tannin turmeric	1
Tannin turmeric	1
Tannin iron oxide	2
Turmeric iron oxide	2

The reasons for the small number of colorants found could be that some of these cloths were prepared specifically as gifts for visitors and the colours and materials that the makers knew would retain their integrity over time were chosen; these colourants were used in prestigious cloths which were always intended to be gifts or for ceremonial uses and crucially like artisans worldwide experience had taught them that some colourants although initially bright and vivid quickly faded and so were rarely used after this discovery. The sequential extraction procedure showed that noni root was used to produce colourants based on morindin and morindone. The colour produced from noni is a stable and true red as opposed to the more brown/red produced from tree bark tannins and therefore noni has been frequently used to create a red colour.

Some colours may have faded so the cloths no longer appeared coloured and perhaps for that reason they were not sampled. In other cases the dye residue may not have been detectable. A number of cloths produced no evidence of colorant but some which appeared pale cream/yellow and seemed uncoloured when analysed showed the presence of turmeric. This may be because of a low application level or the instability of turmeric which is known to fade significantly due to light [15]. Anthocyanins, a widely suggested group of colorants, produce a variety of vivid colours but their colour is also known to fade. Zaffino et al. [16] reported that analysis of anthocyanin samples artificially aged and thus faded could still be detected.

### Case studies

In this section 6 cloths have been chosen as case studies as they represent the variations in colorants used in the cloths studied and also the way in which a colorant derived from the same source can have its colour changed by the addition of other compounds. Where details of their origins and donation date to the collections are known this is included. Figure 3 shows 6 cloths.

#### EBC 42863

**Fig. 4:**
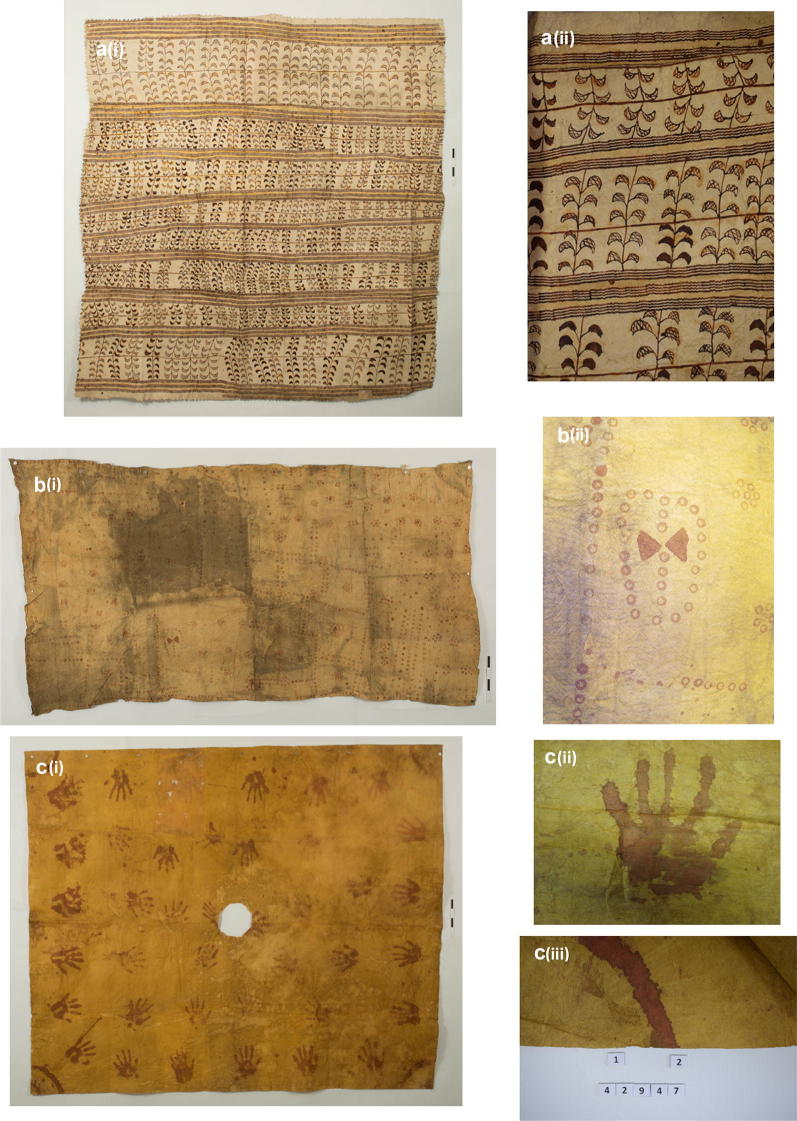
Images of the case studies cloths: EBC 428633 **a** (i) and (ii). E595/1 **b** (i) and (ii). EBC 42947A **c** (i), (ii) and (iii)

The red-brown lines on this leaf patterned cloth (Fig. 4a, i), attributed to Samoa, were too thin to sample but samples were taken from a filled in red-brown leaf (Fig. 4a, ii) and the broad yellow stripe. Both samples showed the presence of turmeric with the red-brown area also containing tannin.

#### Hunterian E595/1

Figure 4b (i) shows a cloth, attributed to Tahiti where a red pattern has been painted on a yellow background. It has considerable areas of soiling perhaps caused by it being folded and the top surface left uncovered. The yellow background is coloured with noni (morinidin) and the red with noni (morindin) plus tannin, detail shown Fig. 4b (ii). This is the only cloth amongst those analysed that used noni to create both the red and yellow colour.

#### EBC 42947(a)

The yellow background of this ‘handprints’ cloth is turmeric (Fig. 4c, i). It is attributed to Hawaii and donated to the EBC in 1874 by HRH the Duke of Edinburgh. Samples obtained from the curved red stripe in the corner found it was painted with noni (morindin). Although no trace of turmeric was detected suggesting these red curves were painted separately and not over the yellow background from the cloth it is difficult to see how this was done (Fig. 4c, ii). It was not possible to sample the red from the hands (Fig. 4c, iii) but detailed study of the area round them suggests that the red was added on top of the yellow background.

#### EBC 42967

**Fig. 5:**
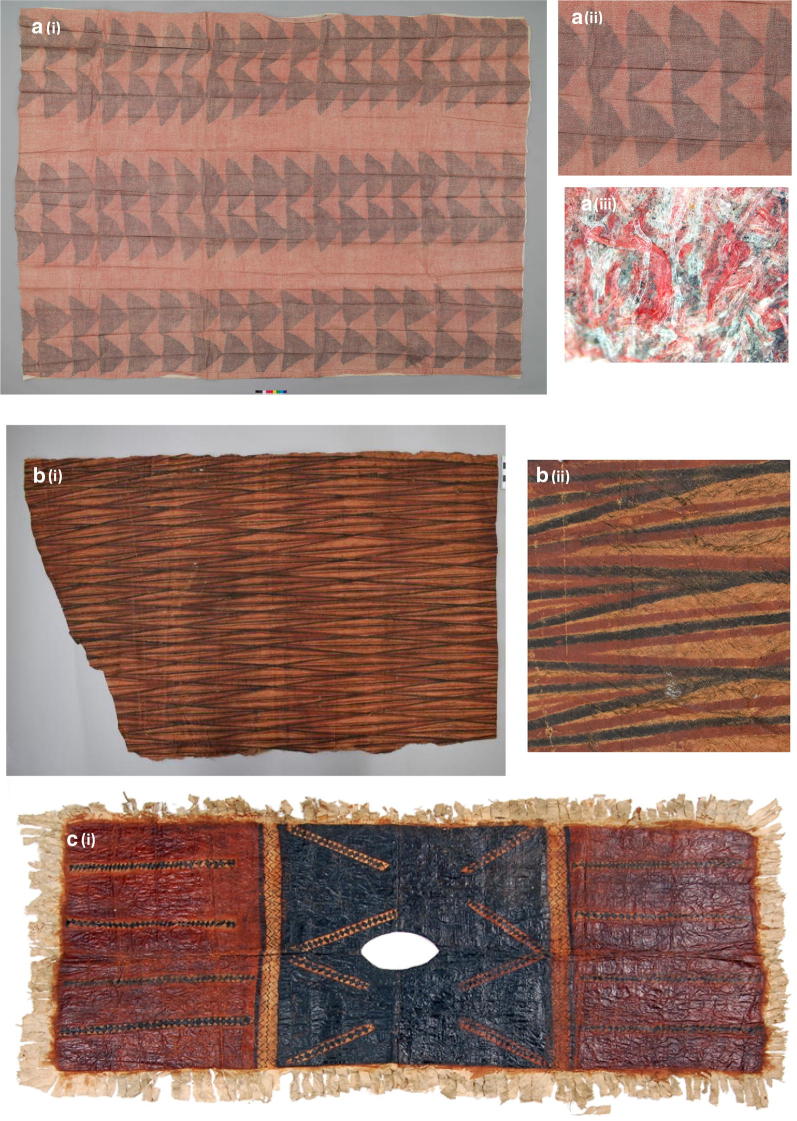
Images of the case studies cloths: EBC 42967 **a** (i), (ii) and (iii). E602 **b** (i) and (ii). EBC 42861 **c** (i)

This cloth (Fig. 5a, i) attributed to Hawaii and donated to the EBC in 1874 by HRH Duke of Edinburgh. The purple and red areas both showed the presence of alizarin and purpurin indicating natural madder, Fig. 5a (ii). Light microscopy of an area (Fig. 5a, iii) shows the presence of coloured fibres not associated with barkcloth fibres. In the nineteenth century the introduction of dyed cloths to the Pacific Islands through trading enabled makers to incorporate these dyed cloths into the beaten barkcloths. “Turkey cloth” and “Turkey red” are cited as a favoured material used in kapamaking [17, 18]. Arthur et al. [17] describes how the Turkey red cotton fabric was shredded and then beaten into bark fibres resulting in a mottled red cloth which was then cut into shapes and beaten into the top layer of the tapa.

Turkey red cloth is a specific type of cotton cloth dyed with madder and mordented with alum and oil; this process was used by European dyers as early as the late eighteenth century [19]. Extraction solution 2 was used for this sample as the dye was likely to contain a mordant and extraction 1 was not sufficiently strong.

#### Hunterian E602

Figure 5b (i) shows this red, yellow and black diamond patterned cloth and Fig. 5b (ii) shows the pattern in more detail. The red and yellow is produced by differing concentrations of iron oxide. There is a trace of turmeric present in both coloured areas. This cloth is attributed to Hawaii whose geology has red earth (ochre). There was no indication that tannins had been used to create mixtures here as HPLC only detected turmeric in very low levels.

#### EBC 42861

Figure 5c shows a poncho/coat type garment ‘worn as a garment by the natives of Samoa’ (http://apps.kew.org/ecbot/specimen/42861), on which the red and black areas contain aluminosilicate, titanium and iron indicating the presence of an iron ochre pigment. The elevated P in the black diamonds and black area may be indicative of soot. Various nuts such as kukui (*Aleurites moluccana*) are burnt and the soot used to create black [6], the soot can be mixed with water, oil or bark tannins. It was not possible to sample this cloth for HPLC analysis so the presence of any additional dye components could not be investigated.

## Conclusions

The findings show that despite the numerous colourant sources for the red, brown and yellow shades listed in the many accounts of historic barkcloth making (Kooijman) only 5 types of plant colorants and 1 inorganic pigment used singly or in combination were identified. A similar finding was reported by Bisulca et al. who examined 150 cloths from the Bishop Museum (Hawaii). The mild analytical extraction procedure prevented decomposition of glycosides allowing for a better understanding of the how these colourants were prepared for use. The case studies show the variations in colour that were achieved by the use of these few colourants, either singularly or in combination.

## Abbreviations

HPLC: high-performance liquid chromatography
XRF: X-ray fluorescence
MS: mass spectrometer
LC–MS: liquid chromatography mass spectrometer
UV–visible: ultra violet–visible
HCl: hydrochloric acid
M: molar
